# Understanding Risk Factors for Oropharyngeal Gonorrhea Among Sex Workers Attending Sexual Health Clinics in 2 Australian Cities: Mixed Methods Study

**DOI:** 10.2196/46845

**Published:** 2024-05-20

**Authors:** Tiffany R Phillips, Christopher K Fairley, Kate Maddaford, Anna McNulty, Basil Donovan, Rebecca Guy, Ruthy McIver, Rebecca Wigan, Rick Varma, Jason J Ong, Denton Callander, Gabrielle Skelsey, Mish Pony, Dylan O'Hara, Jade E Bilardi, Eric PF Chow

**Affiliations:** 1 Monash University Clayton Australia; 2 Alfred Health Melbourne Sexual Health Centre Carlton Australia; 3 Sydney Sexual Health Centre Sydney Australia; 4 The Kirby Institute University of New South Wales Sydney Australia; 5 Operational Centre Geneva Medecins Sans Frontiers Mombasa Kenya; 6 SexTech Lab The New School New York, NY United States; 7 Resourcing Health & Education (RhED) St Kilda Australia; 8 Scarlet Alliance Australian Sex Workers Association Sydney Australia; 9 Vixen Victoria's Peer Sex Worker Organisation Melbourne Australia; 10 Department of General Practice University of Melbourne Melbourne Australia; 11 Centre for Epidemiology and Biostatistics Melbourne School of Population and Global Health University of Melbourne Melbourne Australia

**Keywords:** case-control, qualitative, oral sex, condoms, transactional sex

## Abstract

**Background:**

The risk factors for oropharyngeal gonorrhea have not been examined in sex workers despite the increasing prevalence of gonorrhea infection.

**Objective:**

This study aims to determine the risk factors for oropharyngeal gonorrhea in female and gender-diverse sex workers (including cisgender and transgender women, nonbinary and gender fluid sex workers, and those with a different identity) and examine kissing, oral sex, and mouthwash practices with clients.

**Methods:**

This mixed methods case-control study was conducted from 2018 to 2020 at 2 sexual health clinics in Melbourne, Victoria, and Sydney, New South Wales, Australia. We recruited 83 sex workers diagnosed with oropharyngeal gonorrhea (cases) and 581 sex workers without (controls). Semistructured interviews with 19 sex workers from Melbourne were conducted.

**Results:**

In the case-control study, the median age of 664 sex workers was 30 (IQR 25-36) years. Almost 30% of sex workers (192/664, 28.9%) reported performing condomless fellatio on clients. Performing condomless fellatio with clients was the only behavior associated with oropharyngeal gonorrhea (adjusted odds ratio 3.6, 95% CI 1.7-7.6; *P*=.001). Most participants (521/664, 78.5%) used mouthwash frequently. In the qualitative study, almost all sex workers reported kissing clients due to demand and generally reported following clients’ lead with regard to kissing style and duration. However, they used condoms for fellatio because they considered it a risky practice for contracting sexually transmitted infections, unlike cunnilingus without a dental dam.

**Conclusions:**

Our study shows that condomless fellatio is a risk factor for oropharyngeal gonorrhea among sex workers despite most sex workers using condoms with their clients for fellatio. Novel interventions, particularly targeting the oropharynx, will be required for oropharyngeal gonorrhea prevention.

## Introduction

### Background

There has been an increase in gonorrhea incidence in several countries over the past decade [1-3], which is of particular concern given increasing antimicrobial resistance (AMR) [4,5]. The oropharynx has been implicated in *Neisseria gonorrhoeae* transmission, possibly through saliva exchanged during oral sex practices and tongue kissing [6-9]. The oropharynx is considered a crucial site for AMR given difficulties in treating oropharyngeal gonorrhea and the increased propensity for *N gonorrhoeae* to develop resistance in the oropharynx compared to other anatomical sites [4,10,11]. Sex workers have been identified as an important priority population that bears a disproportionate burden of gonorrhea worldwide, yet data on the prevalence of oropharyngeal gonorrhea in this population are sparse [12]. Therefore, there is an urgent need for research into the risk factors for oropharyngeal gonorrhea, particularly among sex workers, given its importance in AMR [13]. In 2017, an Australian study of female sex workers attending a sexual health clinic in Melbourne, Victoria, Australia, found that female sex workers had a higher prevalence of oropharyngeal gonorrhea (2%) than of genital gonorrhea (1%) [14]. That study also found that oropharyngeal infection was often independent of genital infection [14]. A retrospective study of 42 Australian sexual health clinics showed a 200% increase in oropharyngeal gonorrhea among female sex workers from 2009 to 2015 [15]. Among gay, bisexual, and other men who have sex with men, a case-control study found that oropharyngeal gonorrhea was associated with the number of kissing and receptive fellatio casual sex partners in the preceding 3 months [9]. Due to the overlapping nature of tongue kissing and oral sex practices, it was not possible to undertake an adjusted analysis to determine the risk for any one practice in isolation for the transmission of oropharyngeal gonorrhea [9]. To our knowledge, there has been no similar case-control study conducted to identify risk factors for oropharyngeal gonorrhea in sex workers.

A 2018 study of 180 female sex workers attending a sexual health clinic in Melbourne found that 149 (83.7%) tongue kissed and 175 (97.2%) performed fellatio on at least one male client in an average working week [16]. In addition to the lack of data on risk factors for oropharyngeal gonorrhea among sex workers, there is a lack of qualitative data exploring how sex workers decide whether to engage in tongue kissing and oral sex practices (ie, cunnilingus and fellatio) with male clients. There has been considerable research indicating an increased demand by male clients of female sex workers for the “girlfriend experience,” wherein sex workers and clients engage in sexual practices typical of those in intimate, noncommercial relationships (eg, kissing and cunnilingus) [17,18]. Research on the girlfriend experience tends to focus on the male clients who seek this interaction rather than on the sex workers who provide the service, with no studies exploring the decision to provide these services. A Finnish qualitative study (2008) explored sexual pleasure among female sex workers, a factor very rarely considered in the literature [19]. The study found that, while sex work sometimes required mental distancing or disengagement from the work, which decreases sexual pleasure, the work may empower women by giving them control over the sexual experience and enhancing their own pleasure. No study to our knowledge has explored which factors, including pleasure, may influence the decision of sex workers to engage in kissing and oral sex practices with clients.

Since the mid-2010s, the role of mouthwash has been and continues to be investigated as a novel intervention for gonorrhea prevention in at-risk populations such as men who have sex with men [20-23]. Using mouthwash as a means for harm reduction has been previously recommended for sex workers to reduce sexually transmitted infections (STIs) [24], and a Melbourne-based study has shown that 83% of female sex workers reported using mouthwash daily or weekly [16]. However, it is unclear why sex workers used mouthwash, be it driven by hygiene or a desire to reduce STI transmission, though recent research has shown that Listerine use does not reduce reinfection with gonorrhea [22,23]. In the event that recommendations are made incorporating mouthwash into an STI prevention strategy for sex workers, it would be beneficial to better understand what is driving mouthwash use among sex workers.

### Objectives

The primary aim of this study was to identify the risk factors for oropharyngeal gonorrhea among sex workers (including female and nonbinary sex workers) using a case-control study design. The secondary aim was to explore why sex workers engage in tongue kissing and oral sex and use mouthwash using a qualitative approach.

## Methods

### Study Design

The Health Research in Sex Workers (HERS) study was a mixed methods study comprising an unmatched case-control study and semistructured interviews (Multimedia Appendix 1). Topics from the quantitative data were expanded on and explored in more detail in the semistructured interviews. The interviews were conducted simultaneously with the case-control survey and sought to clarify how and why sex workers may engage in oral sex practices involving saliva with clients.

### Study Setting

The HERS case-control study was conducted at two sexual health clinics in Australia: (1) Melbourne Sexual Health Centre (MSHC) in Melbourne, Victoria; and (2) the Thai and Chinese clinics at Sydney Sexual Health Centre (SSHC) in Sydney, New South Wales (NSW). Participants were recruited for the case-control study at MSHC from November 2018 to March 2020 and at SSHC from November 2018 to December 2019. Both recruitment sites are large public sexual health clinics providing free sexual health services in inner urban settings. Sex work in Australia varies in legality by jurisdiction [25]. At the time of writing (January 2022), sex work was regulated by the Sex Work Act 1994 in Victoria [26], which included the criminalization of all sexual activities that include oral, vaginal, or anal penetration without a condom. In the state of NSW, sex work is decriminalized, there is no legislation criminalizing condomless sex, and risk is managed through Work Health and Safety legislation. High rates of condom use among female sex workers with male clients in both states have previously been reported, including >90% and >78% consistent use for vaginal sex and fellatio in an average working week, respectively [16,27-29].

During the study period, sex workers were required by law to receive mandatory HIV and TI testing every 3 months in Victoria [30]. There is no mandatory HIV and STI testing in NSW; rather, STI testing frequency recommendations are based on individual risk as per the Australian STI Management Guidelines [31].

Participants for the qualitative study were recruited from the MSHC from March 2019 to January 2020. Participants were unable to be recruited from the SSHC due to practical constraints as the interview team was based in Melbourne.

Female sex workers (cisgender and transgender) and sex workers who selected a different identity who were assigned male at birth, were aged ≥18 years, attended either clinic during the study period, and were working in the sex industry at the time of consultation were eligible for the HERS case-control study, and those English-speaking sex workers who attended the MSHC were also eligible for the semistructured interviews.

### Case-Control Study

#### Participants

Sex workers attending the MSHC and SSHC for STI screening had a clinician-collected oropharyngeal swab to test for *N gonorrhoeae*. Cases were defined as sex workers with a positive oropharyngeal swab for gonorrhea, and controls were sex workers who had a negative oropharyngeal swab for gonorrhea.

#### Recruitment

Eligible and interested sex workers attending the MSHC or the Thai and Chinese clinics at the SSHC were given a paper-based questionnaire with a prelabeled study ID by the recruiting clinicians (Multimedia Appendix 2). In addition, sex workers attending either clinic for treatment of oropharyngeal gonorrhea were approached by a research nurse if they had not already completed the questionnaire and asked if they would like to participate in the study. In this way, cases were purposively recruited.

#### Data Collection

The questionnaire was designed by the study investigators with feedback sought from organizations that provide services for sex workers (Resourcing Health & Education [RhED] and the Sex Workers Outreach Project [SWOP], both services for sex workers in Victoria and NSW, respectively). The questionnaire was offered in English, Thai, and Chinese at both recruitment sites (translated by a professional translation company but checked for readability by bilingual clinicians). Participants’ unique patient identifier was recorded on a separate log with the corresponding questionnaire study ID. This log was collected daily by the research staff. Consent was implied by questionnaires being returned completed. No payment was given to participants for returning the questionnaire.

The HERS case-control quantitative questionnaire collected data on demographic characteristics (eg, age, sex, country of birth, length of time in Australia, and languages spoken at home), mouthwash practices, location of sex work, sex practices performed with male clients in an average working week, and sex practices with noncommercial male sexual partners in the previous 7 days. Sex practices with female partners were not ascertained given that most clients are male and the risk of gonorrhea among women who have sex with women is lower than among those who have sex with men [32]. Sex practices included tongue kissing, oral sex (fellatio with and without ejaculation and cunnilingus), vaginal sex, anal sex, sex involving toys, and using saliva (either theirs or a partner’s) as lubricant during sex (vaginal sex, anal sex, and sex involving toys).

Gonorrhea was diagnosed using nucleic acid amplification tests performed on the Aptima Combo 2 assay (Hologic) at the MSHC and the Roche cobas CT and NG assay (Roche Diagnostics) at the SSHC. The Aptima and Roche assays have similar sensitivity and specificity for oropharyngeal gonorrhea detection [33]. Returned questionnaires were linked (via the questionnaire study ID) to the patient identifier number recorded in the log by research staff to match their oropharyngeal gonorrhea test results with their questionnaire data.

#### Study Size

Sample size was calculated using OpenEpi [34] using the estimate that 25% of female sex workers have condomless fellatio [35], an assumed minimum risk factor prevalence of 25% in controls, and a minimum odds ratio (OR) of 2. It should be noted that our original aim was to conduct a 1:4 age-matched case-control study, which would have required 92 cases to 366 controls assuming the same parameters. However, due to the COVID-19 pandemic, recruitment had to be terminated before 92 cases were recruited given that all sex work was required by law to cease during the COVID-19 lockdown nationally (beginning March 25, 2020, in Australia) [36] and because sex practices with clients (and, thus, risk factors for oropharyngeal gonorrhea) might have changed during the pandemic after sex work resumed. For an unmatched case-control study with a ratio of 1:7 cases to controls, at 80% power and a .05 significance, there needed to be 83 cases to 579 controls. There were 83 cases and 581 controls recruited before the COVID-19 lockdown; thus, we were able to conduct an unmatched analysis with 80% power to detect a difference using a significance level of 5%.

This study was reported as per the Strengthening the Reporting of Observational Studies in Epidemiology 2008 guidelines for case-control studies.

#### Statistical Methods

Descriptive statistics were used to calculate the median age of participants. The median number of years in Australia for those born overseas was calculated, and participants were categorized as newly arrived or not based on whether they were in Australia for less than or equal to the median number of years or more than the median number of years. The median was calculated for the number of male clients seen in an average working week. The Mann-Whitney *U* test was used to compare the differences between continuous variables between cases and controls. A chi-square test was used to compare differences in the proportion of categorical variables, and those who declined to report were excluded from the chi-square analysis.

Univariable and multivariable unconditional logistic regression was conducted to identify (1) the factors associated with declining to report sexual practices and (2) the factors associated with oropharyngeal gonorrhea. There were some differences in patient characteristics between the MSHC and SSHC, so we adjusted for site of recruitment in the logistic regression. ORs and the corresponding 95% CIs were reported. Variables with *P*<.10 in the univariable analyses were included in the multivariable analyses. Given the different assays for gonorrhea detection used between the MSHC and SSHC, the site of recruitment was adjusted in the multivariable logistic regression for associations with oropharyngeal gonorrhea. All statistical analyses were conducted using Stata (version 14; StataCorp).

### Qualitative Study

#### Overview

A qualitative descriptive approach was used for the qualitative study component. Qualitative description is a pragmatic rather than theory-driven approach that aims to provide a description of participants’ views and experiences rather than an interpretive, theory-driven analysis [37]. This approach is commonly used in health care when there are specific questions of clinical interest that the study seeks to answer [37].

#### Participants

Sex workers attending the MSHC who completed a HERS English-language version of the questionnaire for the case-control study were eligible for the semistructured interviews.

#### Recruitment

During the recruitment period, participants at the MSHC were shown an invitation at the end of the English-language version of the quantitative HERS questionnaire to participate in a one-on-one semistructured interview on the topics covered in the questionnaire. All participants who responded “yes” to taking part in the interviews were contacted via SMS text message (from the phone numbers listed on their medical records at the MSHC), and interviews were arranged either face-to-face or over the phone, according to participant preference, with those still wanting to participate. Written or verbal (if the interview was conducted over the phone) informed consent was obtained from participants after the study information was discussed with them.

#### Data Collection

The interview schedule was designed by the research team at the MSHC and then reviewed by the ACON Research Ethics Review Committee; the SWOP in NSW; and RhED, a service for sex workers in Victoria.

The interview schedule contained questions regarding sexual practices they performed at work that involved saliva exchange (either theirs or the clients’), as well as their use of mouthwash at work. The interview schedule was based on the case-control survey questions, allowing for further exploration and depth of understanding regarding oral sex practices that involve saliva (specifically, adding depth to the data in terms of the “what,” “how,” and “why” of these practices). Participants were given an Aus $50 (US $32.08) gift voucher after the completion of their interview (either in person or by mail in an unmarked envelope) to reimburse them for their time. All the interviews were audio recorded with the participants’ permission and transcribed verbatim. Transcripts were deidentified. Audio files and transcripts were stored on secure servers and will be destroyed after 7 years in line with Alfred Health ethics requirements.

Interviews were conducted in English by 1 of 2 researchers (TRP and KM), both of whom have experience with conducting interviews on sensitive sexual health issues. TRP sat in on the first 2 interviews conducted by KM, and the 2 researchers reviewed and discussed the interviews afterward to ensure consistency in the approach to them. TRP and KM met weekly to discuss the interviews, the interview schedule, and the developing themes. Upon completion of 12 interviews, the wider qualitative research team members (TRP, KM, and JB) met to discuss the data, the developing themes, and any further lines of questioning required. At this point, one question was added to the interview schedule to further explore a topic that some participants had touched on in the interviews. The new question concerned hypothetical willingness to change sexual practices at work if saliva was shown to transmit gonorrhea.

A total of 19 interviews had been completed when participant recruitment was interrupted by the COVID-19 pandemic. The research team met to discuss preliminary findings; it was decided that recruitment would not continue in late 2020 and 2021 due to the likely differing sex practices and risk perceptions with regard to COVID-19 as well as the ongoing COVID-19 lockdowns and restrictions causing continued disruptions in sex work and research in Australia.

According to Braun and Clarke [38], the concept of data saturation and its meaning and use depend on the purpose and goals of a study and the data analysis approach being used. While Braun and Clarke [38] argue that data saturation is not the best rationale for sample size, particularly when using a reflective thematic data analysis approach, when studies are conducted for largely pragmatic reasons and follow a fairly structured deductive approach to data analysis in which the data generated are relatively concrete, participants are largely recruited from a particular setting, coding largely relates to the broad topics or interview questions, and themes act largely as a summary of participants’ differing responses with little further interpretation regarding meaning, the concept of data saturation, or the point at which no “new” insights are provided, may be useful [38]. Given the deductive nature of the analysis and that the themes were largely guided by the research questions, the concept of data saturation was applied, with themes saturated among a broad sample with a range of ages, years working in the sex industry, and experiences in the context of brothel-based sex work in Melbourne. However, further insights into 2 particular groups of sex workers would have been beneficial, including those who worked outside of brothels or who were cases (diagnosed with oropharyngeal gonorrhea at the time of recruitment). For pragmatic reasons previously outlined (recruitment had to cease due to the COVID-19 pandemic), this was not possible.

#### Data Analysis

Data were analyzed using deductive thematic analysis, a “top down” approach wherein coding and analysis are largely informed by the ideas and concepts the researcher brings to the data rather than being created from the data themselves. In the case of this study, coding and themes were largely guided by the interview schedule questions and topics, which had been informed by literature in the field and the specific questions of clinical interest that the research team sought to answer [39]. Each transcript was initially read and coded by TRP. After all transcripts were coded, they were imported into NVivo (version 12; QSR International) for data management. The codes were grouped and labeled into preliminary themes before each transcript was read again, and the themes were further revised, refined, and compared for similarities and differences. A subset of transcripts was read and coded by JB and KM, after which all 3 researchers (TRP, JB, and KM) met to discuss and reach a final consensus on the themes. There were no major differences in interpretation evident.

This study is reported as per the RATS guidelines for qualitative research [40].

### Ethical Considerations

This study was approved by the Alfred Hospital Ethics Committee, Melbourne, Victoria, Australia (project 596/17), and the South Eastern Sydney Local Health District (reference: 18/G/166). This study was also approved by the ACON Research Ethics Review Committee (reference number: 2018/17) with support from the SWOP NSW. This project was also reviewed by RhED, a service for sex workers in Victoria. As stated above, informed consent was given for the interviews and implied consent was considered by a returned completed questionnaire for the case-control study. Data presented is anonymous. Participants in the qualitative interviews were compensated Aus $50 (US $32.08). There was no compensation for participants in the case-control study.

## Results

### Case-Control Study

There were 386 surveys included from the MSHC, of which 23 (6%) were from cases and 363 (94%) were from controls. There were 278 surveys included from the SSHC, of which 60 (21.6%) were from cases and 218 (78.4%) were from controls (Multimedia Appendix 2). Thus, of the 664 surveys, the total number of cases was 83 (12.5%), and the total number of controls was 581 (87.5%). Of the 664 surveys included in the analysis, there were 542 (81.6%) that were returned with every section completed, 12 (1.8%) that were returned with every section except the demographics and mouthwash sections completed, and 110 (16.6%) that were returned with every section except the sex practice with clients and noncommercial partners sections completed. Being born in China or other countries aside from Thailand was associated with returning the survey with incomplete sections for sexual practices (Multimedia Appendix 3).

The median age of participants was 30 (IQR 25-36) years, and participants at the SSHC were older than those from the MSHC (median age 33 vs 28 y; *P*<.001; Table 1). Cases were older than controls (median age 32 vs 30 y; *P*=.01).

**Table 1:** Risk factors for oropharyngeal gonorrhea among female sex workers recruited from 2 sexual health clinics in Australia (N=664).

				Cases (n=83), n (%)		Controls (n=581), n (%)		OR^a^ (95% CI)		aOR^b^ (95% CI)		*P* value for aOR
**Site**												
	MSHC^c^			23 (27.7)		363 (62.5)		Reference		Reference		—^d^
	SSHC^e^			60 (72.3)		218 (37.5)		4.3 (2.6-7.2)^f^		3.1 (1.5-6.2)		.001
**Gender identity**												
	Female			79 (95.2)		531 (91.4)		Reference		—		—
	Transgender and gender diverse			4 (4.8)		50 (8.6)		0.5 (0.2-1.5)		—		—
**Age (y)**												
	≤24			8 (9.6)		121 (20.8)		Reference		Reference		—
	25-34			43 (51.8)		289 (49.7)		2.3 (1.0-4.9)^f^		1.2 (0.5-2.9)		.70
	≥35			32 (38.6)		171 (29.4)		2.8 (1.3-6.4)^f^		1.1 (0.4-2.8)		.85
**Newly arrived in Australia (within 3 years)**												
	No			34 (41)		370 (63.7)		Reference		Reference		—
	Yes			49 (59)		211 (36.3)		2.5 (1.6-4.0)^f^		1.2 (0.6-2.1)		.63
**Smoke daily**												
	No			60 (72.3)		427 (73.5)		Reference		—		—
	Yes			20 (24.1)		141 (24.3)		1.0 (0.6-1.7)		—		—
	Declined to report			3 (3.6)		13 (2.2)		1.6 (0.5-5.9)		—		—
**Use mouthwash^g^**												
	Infrequently			10 (12)		122 (21)		Reference		Reference		—
	Frequently			69 (83.1)		452 (77.8)		1.9 (0.9-3.7)^f^		1.0 (0.5-2.2)		.97
	Did not disclose			4 (4.8)		7 (1.2)		7.0 (1.7-27.9)^f^		11.2 (2.2-57.2)		.004
**Type (venue) of sex work**												
	Brothel only			47 (56.6)		289 (49.7)		Reference		—		—
	Massage parlor only			12 (14.5)		139 (23.9)		0.5 (0.3-1.0)^f^		0.2 (0.1-0.5)		<.001
	Multiple or other venues			24 (28.9)		150 (25.8)		1.0 (0.6-1.7)		1.0 (0.6-1.9)		.90
	Declined to report			0 (0)		3 (0.5)		—		—		—
**Sexual practices with clients**												
	**Tongue kiss male clients^h^**											
		No	9 (10.8)		146 (25.1)		Reference		Reference		—	
		Yes	51 (61.4)		348 (59.9)		2.4 (1.1-5.0)^f^		1.5 (0.7-3.5)		.30	
		Declined to report	23 (27.7)		87 (15)		4.3 (1.9-9.7)^a^		3.3 (1.2-9.4)		.02	
	**Perform condomless fellatio on male clients^h^**											
		No	17 (20.5)		346 (59.6)		Reference		Reference		—	
		Yes	48 (57.8)		144 (24.8)		6.8 (3.8-12.2)^f^		3.6 (1.7-7.7)		.001	
		Declined to report	18 (21.7)		91 (15.7)		4.0 (2.0-8.1)^f^		1.1 (0.8-6.0)		.14	
	**Client ejaculated in mouth^h^**											
		No	43 (51.8)		440 (75.7)		Reference		Reference		—	
		Yes	25 (30.1)		56 (9.6)		4.6 (2.6-8.0)^f^		1.4 (0.7-2.9)		.32	
		Declined to report	15 (18.1)		85 (14.6)		1.8 (1.0-3.4)^f^		0.6 (0.3-1.5)		.30	
**Sexual practices with not-at-work sexual partners**												
	**Tongue kiss noncommercial sexual partner^i^**											
		No^j^	48 (57.8)		341 (58.7)		Reference		Reference		—	
		Yes	20 (24.1)		182 (31.3)		0.8 (0.4-1.4)		1.4 (0.7-2.7)		.30	
		Declined to report	15 (18.1)		58 (10)		1.8 (1.0-3.5)^f^		1.6 (0.5-5.4)		.47	
	**Performed condomless fellatio on not-at-work sexual partner^i^**											
		No^j^	57 (68.7)		397 (68.3)		Reference		—		—	
		Yes	8 (9.6)		104 (17.9)		0.5 (0.2-1.2)		—		—	
		Declined to report	18 (21.7)		80 (13.8)		1.6 (0.9-2.8)		—		—	
	**Not-at-work sexual partner ejaculated in mouth^i^**											
		No^j^	68 (81.9)		453 (78)		Reference		Reference		—	
		Yes	1 (1.2)		70 (12)		0.1 (0.0-0.7)^f^		0.1 (0.0-0.6)		.02	
		Declined to report	14 (16.9)		58 (10)		1.6 (0.9-3.0)		0.9 (0.3-3.1)		.88	

^a^OR: odds ratio.

^b^aOR: adjusted odds ratio.

^c^MSHC: Melbourne Sexual Health Centre.

^d^Not applicable.

^e^SSHC: Sydney Sexual Health Centre.

^f^Variables with *P* values of <.10 in the univariable analyses were included in the multivariable analyses.

^g^Infrequent mouthwash use included those who selected *never*, *yearly*, or *monthly*. Frequent mouthwash use included those who selected *daily* or *weekly*.

^h^Participants were asked to report any sex practices with male clients in an average working week.

^i^Participants were asked to report sex practices with not-at-work male partners in the previous 7 days.

^j^Includes 328 controls and 47 cases who did not have a noncommercial sex partner.

There were 8.1% (54/664) transgender and gender-diverse participants. Most participants were born in Australia or New Zealand (230/664, 34.6%), followed by Thailand (166/664, 25%), China (156/664, 23.5%), and other countries (112/664, 16.9%). Overall, there were 39.2% (260/664) of participants who were newly arrived in Australia. For those cases who were born outside Australia, the median length of time in Australia was 2 (IQR 1-3) years compared to 3 (IQR 2-6) years for controls (*P*=.002). There was no difference in the proportion of transgender and gender-diverse participants between the 2 sites (28/386, 7.3% from the MSHC vs 26/278, 9.4% from the SSHC; *P*=.33). Most participants from the SSHC were born in Thailand (147/278, 52.9%), China (120/278, 43.2%), or Taiwan (5/278, 1.8%); however, there were 2.2% (6/278) who were born in other countries. Most participants from the MSHC were born in Australia or New Zealand (228/386, 59.1%), followed by China (36/386, 9.3%), Thailand (21/386, 5.4%), India (7/386, 1.8%), and Malaysia (7/386, 1.8%). The remaining 22.5% (87/386) of participants from the MSHC were from 39 different countries. For those cases born outside Australia, the median length of time in Australia was 2 (IQR 1-3) years compared to 3 (IQR 2-6) years for controls (*P*=.002).

Most participants worked in only one type or venue of sex work; most worked at brothels only (336/664, 50.6%), followed by massage parlors (151/664, 22.7%) and private (72/664, 10.8%) and street-based sex work (1/664, 0.2%). There were 15.1% (100/664) of participants who worked in more than one type or venue of sex work, of which the most common combination was brothel and private sex work (56/100, 56%).

Of the 578 participants who answered the question on number of male clients in an average working week, the median was 10 (IQR 6-20) clients; there was no significant difference in the number of clients between cases and controls (*P*=.18; Table 2). There were no significant differences in the proportion who performed fellatio on clients, received cunnilingus from clients, had vaginal or anal sex with clients, or had sex involving sex toys between cases and controls (Table 2). However, a significantly higher proportion of cases tongue kissed their clients (51/60, 85% vs 348/494, 70.4%; *P*=.02), performed condomless fellatio on clients (48/65, 74% vs 144/490, 29.4%; *P*<.001), and had condomless vaginal sex (17/60, 28% vs 78/470, 16.6%; *P*=.03) and condomless anal sex (10/18, 56% vs 56/183, 30.6%; *P*=.03) with clients in an average working week.

**Table 2:** Number of male clients seen in an average working week among female sex workers by cases with oropharyngeal gonorrhea and controls (N=664).

			Cases (n=83)		Controls (n=581)		*P* value^a^	
Number of male clients seen in an average working week, median (IQR)^b^			10 (5-15)		10 (6-20)		.18	
**Tongue kissed male clients in an average working week, n (%)**								.02
	No	9 (10.8)		146 (25.1)				
	Yes	51 (61.4)		348 (59.9)				
	Declined to report	23 (27.7)		87 (15)				
Number of clients tongue kissed, median (IQR)			2 (0-9)		2 (0-6)		.17	
Proportion of clients tongue kissed (%), median (IQR)^c^			40 (10-90)		50 (10-80)		.43	
**Perform fellatio on male clients in an average working week, n (%)**								.05
	No	2 (2.4)		54 (9.3)				
	Yes	63 (75.9)		447 (76.9)				
	Declined to report	18 (21.7)		80 (13.8)				
Number of clients performed fellatio on, median (IQR)			5 (2-9)		7 (2-14)		.12	
Proportion of clients performed fellatio on (IQR)^c^			90 (50-100)		70 (30-99)		.02	
**Perform condomless fellatio on male clients in an average working week, n (%)**								<.001
	No	17 (20.5)		346 (59.6)				
	Yes	48 (57.8)		144 (24.8)				
	Declined to report	18 (21.7)		91 (15.7)				
Number of clients performed condomless fellatio on, median (IQR)			1 (0-3)		0 (0-0)		<.001	
Proportion of clients performed condomless fellatio on (IQR)^c^			50 (40-80)		50 (20-90)		.29	
**Receive cunnilingus from male clients in an average working week, n (%)**								.14
	No	5 (6)		75 (12.9)				
	Yes	57 (68.7)		422 (72.6)				
	Declined to report	21 (25.3)		84 (14.5)				
Number of clients received cunnilingus from, median (IQR)			3 (1-7)		4 (1-8)		.62	
Proportion of clients received cunnilingus from (%), median (IQR)^c^			40 (10-50)		50 (20-70)		.08	
**Vaginal sex with clients in an average working week, n (%)**								.24
	No	9 (10.8)		54 (9.3)				
	Yes	45 (54.2)		428 (73.7)				
	Declined to report	29 (34.9)		99 (17)				
Number of clients had vaginal sex with, median (IQR)			5 (2-15)		8 (3-15)		.11	
Proportion of clients had vaginal sex with (IQR)^c^			90 (60-100)		95 (80-100)		.69	
**Condomless vaginal sex with clients in an average working week, n (%)**								.03
	No	43 (51.8)		392 (67.5)				
	Yes	17 (20.5)		78 (13.4)				
	Declined to report	23 (27.7)		111 (19.1)				
Number of clients had condomless vaginal sex with, median (IQR)			0 (0-0)		0 (0-0)		.001	
Proportion of clients had condomless vaginal sex with (IQR)^c^			20 (20-70)		50 (10-90)		.37	
**Anal sex with clients in an average working week, n (%)**								.40
	No	45 (54.2)		395 (68)				
	Yes	6 (7.2)		77 (13.3)				
	Declined to report	32 (38.6)		109 (18.8)				
Number of clients had anal sex with, median (IQR)			0 (0-0)		0 (0-0)		.92	
Proportion of clients had anal sex with (%), median (IQR)^c^			35 (5-80)		10 (5-30)		.21	
**Condomless anal sex with clients in an average working week, n (%)**								.03
	No	8 (9.6)		127 (21.9)				
	Yes	10 (12)		56 (9.6)				
	Declined to report	65 (78.3)		398 (68.5)				
Number of clients had condomless anal sex with, median (IQR)^c^			0 (0-0)		0 (0-0)		.37	
Proportion of clients had condomless anal sex with (IQR)^c^			100 (100-100)		100 (99-100)		N/A^d^	
**Use sex toys with clients in an average working week, n (%)**								.19
	No	41 (49.4)		321 (55.2)				
	Yes	12 (14.5)		147 (25.3)				
	Declined to report	30 (36.1)		113 (19.4)				
Number of clients had sex with involving toys, median (IQR)			0 (0-0)		0 (0-0)		.57	
Proportion of clients had sex with involving toys (IQR)^c^			20 (10-50)		10 (5-20)		.14	

^a^*P* values were calculated excluding those who declined to report each practice using the chi-square test for categorical variables and Mann-Whitney *U* test for continuous variables.

^b^There were 15 cases and 71 controls who declined to report the number of clients and, thus, were excluded from this analysis.

^c^Partner number for each activity was calculated by multiplying the proportion of clients with whom they performed each activity by the total number of clients seen in an average working week.

^d^N/A: not applicable.

There was no difference in the total number or proportion of clients with whom they engaged in each sexual activity between cases and controls (Table 2) except that cases reported fewer clients on whom they performed fellatio (median 5, IQR 3-10) than controls (median 9, IQR 4-14; *P*=.006; Table 2). However, cases had performed condomless fellatio on more clients than controls (median 3, IQR 0-6 for cases and median 0, IQR 0-1 for controls; *P*<.001).

In the multivariable logistic regression, after adjusting for recruitment site, age, length of time in Australia, frequency of mouthwash use, tongue kissing male clients, performing condomless fellatio on male clients, whether clients ejaculated in their mouth, tongue kissing noncommercial sexual partners, and whether noncommercial sexual partners ejaculated in their mouth, the only sex practice that was a risk factor for oropharyngeal gonorrhea was performing condomless fellatio on male clients (adjusted OR 3.6, 95% CI 1.7-7.6; *P*=.001; Table 1).

There were 22.6% (120/530) of participants who believed that mouthwash could prevent them from acquiring STIs in the throat, 42.5% (225/530) who did not think that mouthwash could prevent STIs in the throat, and 34.9% (185/530) who said that they did not know. There was no significant difference among those who believed that mouthwash could prevent STIs in the throat between cases and controls (*P*=.26).

### Qualitative Findings

#### Overview

In total, 9.5% (63/664) of sex workers indicated interest in participating in the interviews and were contacted by a research nurse at the MSHC. When contacted, of the 63 sex workers, 28 (44%) at the MSHC agreed to participate and scheduled a time for the interview; however, 9 (32%) did not attend their scheduled interview and could not be reached to reschedule. In total, 19 participants completed interviews before data collection was discontinued. The age of the 19 participants ranged from 18 to 44 years, with a median of 28 (IQR 24-31) years (Table 3). The duration of interviews ranged from 24 to 61 minutes, with a median of 41 minutes. One participant in the qualitative interviews was a case in the case-control group, whereas the rest were from the control group.

**Table 3:** Participant demographics for the qualitative interviews among female sex workers recruited from the Melbourne Sexual Health Centre (N=19).

		Participants, n (%)
**Gender identity**		
	Woman	18 (95)
	Nonbinary	1 (5)
**Oropharyngeal gonorrhea**		
	Case	1 (5)
	Control	18 (95)
**Age range (y)**		
	18-24	7 (37)
	25-30	6 (32)
	31-35	6 (32)
**Length of time in the sex industry (y)**		
	≤1	3 (16)
	2	3 (16)
	≥3	13 (68)
**Country of birth**		
	Australia or New Zealand	17 (89)
	Overseas	2 (11)
**Location of sex work**		
	Brothel	16 (84)
	Massage parlor	1 (5)
	Private	1 (5)
	Brothel and private	1 (5)
**Mouthwash use at work**		
	Before and after clients	8 (42)
	Before clients	4 (21)
	After clients	3 (16)
	Rarely or never	4 (21)

The qualitative data were organized into six descriptive themes related to the kissing and oral sex practices and mouthwash use among sex workers (Textbox 1): (1) the “how” and “why” of kissing clients, (2) always covered—fellatio with clients, (3) not so risky—uncovered cunnilingus from clients, (4) clients’ saliva as lubricant is a “no go,” (5) pleasure with clients—a “perk” or not part of the job, and (6) mouthwash use with clients—a freshener and germ killer.

**An overview of female sex worker practices—the what, the why, and the how from semistructured interviews with 19 female sex workers recruited from the Melbourne Sexual Health Centre.**

**Kissing (theme 1: the “how” and “why” of kissing clients)**

- Very common in some form
- Sex worker initiated
  - Typically closed mouth or shallow
  - To improve relationship with regular clients
  - To facilitate a more intimate experience
  - Pressured by client demand and availability
- Client initiated
  - Often negotiated before encounter
  - Commanded higher premium
- Expectations for clients
  - Good oral hygiene
  - Sobriety

**Performing oral sex (fellatio; theme 2: always covered—fellatio with clients)**

- Very common
- Condoms used always
  - Perceived as “high risk”
  - Reduce sexually transmitted infection (STI) risks
  - Legal requirements
  - Public health campaigns
- Client pressure
  - Financial incentive
  - Waiting until the encounter had begun

**Receiving cunnilingus (theme 3: not so risky—uncovered cunnilingus from clients)**

- Very common
- Dental damns never used
  - Perceived as “low” or “no risk”
  - Detracts from client experience
  - Difficult to use
  - Not common or industry standard
  - No public health campaigns

**Saliva as lubricant (theme 4: clients’ saliva as lubricant a “no go”)**

- Uncomfortable with client using saliva as lubricant
  - Perceived as “high risk”
  - Disgusted by the idea
  - Not as effective as actual lubricant
- May sometimes use their own saliva as lubricant
  - Not preferred over actual lubricant
  - Will use out of convenience

**Pleasure with clients (theme 5: pleasure with clients—a “perk” or not part of the job)**

- Does not influence which sex practices are engaged in
- Some feel that it is not possible to feel pleasure with clients
  - Compartmentalizing as “work” and not for enjoyment
  - Lack of emotional connection
- Pleasure infrequent but can impact kissing and cunnilingus
  - May kiss for longer or deeper
  - May allow client to kiss or perform cunnilingus despite not paying for the service
- More enjoyment over time
  - More confident and relaxed with clients

**Using mouthwash (theme 6: mouthwash use with clients—a freshener and germ killer)**

- Routinely used by some female sex workers
  - Freshen up before the booking
  - Kill any germs they may have picked up from the client after the booking
  - As a matter of routine to “clean the slate” before the next client
- Occasionally used by some female sex workers
  - When they feel their breath needs to be freshened up before a booking
  - After a booking if left with unclean feeling
- Some never use mouthwash at work
  - Believe it is bad for oral health
- Most prefer that clients use mouthwash before the booking
  - Freshen breath
  - Perception that it makes client less likely to transmit germs
  - Brothel provides mouthwash
  - Expectation that client uses mouthwash and showers before the booking

#### The How and Why of Kissing Clients

All participants in the qualitative interviews (19/19, 100%) reported kissing at least some of their clients; however, the style of kissing varied from closed-mouth kissing (“pecks”) and shallow tongue kissing to deep tongue kissing. For most participants, whether they kissed clients depended on whether the client requested the service before the booking as it entails an additional cost, although there were a few participants who reported reserving kissing for regular clients, those they judged to have good oral hygiene (ie, clean teeth), or those not visibly intoxicated.

The style and duration of kissing was most often dictated by the client’s preference and initiation, with almost half of participants describing following the clients’ lead:

So, it’s because they’re paying for it, it’s how they’re wanting that to happen. I’ll generally—if I’m doing the instigation of the kissing, it will usually be more pecking than full tongue. But there will still be saliva on the lips and that sort of thing. Then it’s up to them how they want to take it further. I continue on the line that they’re taking things.

Participant 5; aged 30 years; less than a year in the sex industry; brothel based

Other participants did not allow the client to dictate kissing style; instead, they reported efforts to avoid deep kissing with clients, such as kissing with closed teeth or telling the client to use less tongue. Some described general discomfort with deep kissing clients, including one who felt that it was too intimate for work:

I just find the sharing of that much saliva is a bit...too...it’s a bit too intimate for just my work...

Participant 1; aged 24 years; 4 years in the sex industry; massage parlor

While participants commonly stated that pay was the only factor in deciding to kiss a client or not, there were several who felt pressured to agree to kiss clients to compete with other sex workers for clients. However, a couple of participants felt that kissing clients enhanced the booking in terms of making the service feel more genuine as well as allowing them to engage with the client in a more natural way.

#### Always Covered: Fellatio With Clients

All participants (19/19, 100%) described performing oral sex (fellatio) on some clients, and all (19/19, 100%) reported always using condoms for this practice. Almost all participants reported that the main reason for using condoms during fellatio was for safety as they did not want to contract an STI in the throat, whereas almost half of participants reported using condoms due to the legal requirement. One participant shared the following:

Because it’s the law and I want to be really safe. That’s not—if I—I’ve had many, many, many people try to get it without a condom, but I don’t know these people. I don’t know where they’ve been. I need that protection.

Participant 9; aged 18 years; less than a month in the sex industry; brothel based

Many of these participants described seeing posters in their workplaces that caution against performing fellatio on clients without a condom. Some participants reported receiving pressure from clients to perform oral sex without a condom, most often after the booking had commenced and they were alone in the room. Several participants reported men offering to pay more for a “natural” (condomless) fellatio service, and while reportedly declining these requests, several also said that they knew of other sex workers in their workplaces who accept these offers for more pay.

#### Not So Risky: Uncovered Cunnilingus From Clients

All participants except one reported having some clients perform oral sex (cunnilingus) on them, and none reported using a dental dam for this practice. Reasons for not using a dental dam varied, but the common sentiment among participants was that cunnilingus did not seem to place them at as much risk of STIs compared to them performing condomless fellatio on clients:

I’ve heard it’s [cunnilingus] more likely to affect them more than me. That’s what I’ve been told anyway. So it’s like their risk, not mine and I know I’m clean, because I get tested all the time.

Participant 6; aged 26 years; 6 years in the sex industry; brothel based

Other reasons for not using a dental dam revolved around the dental dam detracting from the experience for the client. Many participants described dental dams as being difficult to use and anticipated that enforcing clients to use one would deter them from booking services with them and, ultimately, lead to less bookings. Several participants commented that it was not industry standard to use dental dams and there were no posters warning sex workers to use them for oral sex like there were for condoms.

#### Client’s Saliva as a Lubricant a “No Go”

With few exceptions, almost all participants were uncomfortable having a client use their own saliva as lubricant for sex, though several described previously having clients spit on their hands and touch the participants’ genitals without asking first and before they could be stopped. Reasons for not being comfortable included a fear of contracting STIs from the client’s saliva, preferring actual lubricant over saliva in general as it is more effective, and being generally disgusted by the client’s saliva. One participant said the following:

Um, it’s [client’s saliva] kind of gross I guess it’s kind of a bit sticky and not very, yeah, not very effective.

Participant 4; aged 21 years; less than a year in the sex industry; brothel based

In general, most participants described preferring actual lubricant during sex for its effectiveness over using their own saliva as lubricant with clients. However, there were several participants who were comfortable using their own saliva on occasion. Among these participants, saliva was only used as lubricant because of the convenience, with most still preferring lubricant if it was on hand.

Participants were not explicitly asked to reflect on why they might be comfortable with a client performing cunnilingus on them without a dental dam but not having a client use their saliva as a lubricant for vaginal sex. However, several participants raised this of their own accord. For one participant, the reasoning for this was because the saliva used as a lubricant would go inside her body, whereas she imagined that the client’s saliva during cunnilingus is not necessarily “inserted” into her vagina.

#### Pleasure With Clients: A “Perk” or Not Part of the Job

Most participants felt that their pleasure in a booking would not influence their decision to engage in oral sex practices with clients but rather was seen as a fringe benefit:

No, not really. If I’m enjoying it, then I’m enjoying it, and that’s just a lucky perk. I wouldn’t stray from my boundaries just because they’ve kind of sprung something on me or whatnot. My service is my service and I stick to it.

Participant 12; aged 26 years; 8 years in the sex industry; brothel based

Of these participants who said that pleasure was not a factor in their decision to engage in oral sex practices with clients, several felt that actually feeling pleasure from sex work was not possible as they compartmentalized it as work rather than for enjoyment. Others felt that pleasure during sex work was not possible due to the lack of emotional connection:

I find that I, um, even if I have like the best client in the world, I’m never gonna, like have a pleasurable experience at work, because you’re always just like, on your guard a little bit, and you’re, I’m at work, you know...like I’m like just totally zoned out (laughs) Like they don’t think that, but I’m like thinking about what I’m going to eat for dinner, and, just like whatever, and so...I’ll never be like “I-I really wanna do this thing, and like get pleasure out of this” that will never happen...But, I will definitely make choices to avoid, like, things that I know I definitely don’t like.

Participant 2; aged 26 years; 2 years in the sex industry; brothel based

However, there were several participants for whom their pleasure, while infrequent with clients, could influence the duration or style of sex practices with clients, most notably kissing and cunnilingus (but not condom use). These participants described kissing for longer or deeper if they were enjoying the service with the client and, in some cases, allowing the client to kiss or perform cunnilingus on them if they had not paid for the service or simply to perform cunnilingus on them for longer if it was enjoyable:

...like if it was someone where there was a lot of saliva and it wasn’t really feeling very good, I probably would let them do that for a while and then I’d just sort of be like “oh, it’s your turn now.” But if I’m having a really good time and it’s feeling really great, I’m not going to interrupt, and stop that person from what they’re doing sooner, if it means that I might get to have a really great time or even reach climax. You just kind of go along with it a bit more when it’s like, a really good time. But not to the point where I would, you know, not to the point where I would have unprotected oral sex on them, or oral, or vaginal sex with me.

Participant 14; aged 32 years; 2 years in the sex industry; brothel based

Among the participants who felt that their pleasure sometimes influenced their oral sexual practices with clients, all but one stated that sex work became more enjoyable over time after they became more confident in their work:

[I enjoy it more now] Because I’m more relaxed with my clients, I’m more confident and experienced and I like to connect with them. Whereas before, I was like I don’t want to get to know you, but now I pretty much make friends with all my clients, so it's more enjoyable.

Participant 7; aged 24 years; 4 years in the sex industry; brothel based

#### Mouthwash Use With Clients: A Freshener and Germ Killer

Almost half of the participants reported using mouthwash before and after clients at work as a matter of routine. The main reason for using mouthwash before a client was to freshen up their breath before the booking. The main reason for using mouthwash after a client was to kill any germs they might have gotten from the client during the booking, particularly germs that cause bad breath and the common cold; however, gonorrhea was also occasionally mentioned. The other reason was to “clean the slate” for the next client; using mouthwash helped them feel refreshed and ready to see the next client:

...when I use mouthwash and redo my make-up, it’s like okay, that client’s finished, done with, go out and I’m ready to present again for the next client.

Participant 9; aged 18 years; less than a month in the sex industry; brothel based

The remaining half of participants used mouthwash less routinely and tended to use it before a booking if they felt that they needed to freshen their breath or after a client who was a smoker or had left a feeling of uncleanliness in their mouth. There were only a few participants who never used mouthwash at work, and these participants did not believe mouthwash was good for their oral health.

Most participants preferred clients to use mouthwash before bookings so that they had fresher breath and were less likely to transmit germs. Generally, for these participants, the brothels provide mouthwash and disposable cups for clients to use, and the mouthwash is poured and waiting for them when they enter the room for the booking. In this way, the participant does not have to ask the client to use mouthwash directly, which several described they would feel too uncomfortable doing as it might be considered rude. However, several others reported that they did not hesitate to ask clients to use the mouthwash if they noticed that it had not been used or if their breath smelled.

One participant, the only participant in our interviews who was a case upon recruitment (had oropharyngeal gonorrhea), made clients use a “cocktail” of mouthwash she brought from home for a full minute that combined Betadine, an antibacterial sore throat gargle, with Cepacol, an antibacterial mouthwash, which she used before and after every client as well. This participant had been using this “cocktail” of mouthwash before every client for 2 months before she became infected with oropharyngeal gonorrhea. This participant did not have outside-of-work sexual partners and did not perform fellatio without a condom with clients, nor did she report any other oral sex practices (ie, rimming or spit play) aside from tongue kissing. She felt that her “cocktail” of mouthwash kept her safe from germs that cause colds and gave her the security to engage in “passionate” tongue kissing that she felt gave her a competitive edge over other sex workers:

Like, if I don’t kiss at all, even lips, they will feel like “oh, this girl, she’s terrible, I’m not coming back” Like how can I have a passionate moment if I don’t kiss, you know? I try to trick them, like, oh it’s a passionate kissing without opening my mouth, you know what I mean? But like, if I do kiss with tongue, they love it. Like, they fall in love, they will always come back, and they will tell their friends, so their friends will come. Like most of my regular clients, I get them because of that, that treatment, you know? But obviously I won’t do that to someone I don’t feel comfortable. But it does help me to make a lot of money, you know. Have a lot of clients, and regular clients. And you know what because I do the mouthwashing, I ask them to do, I put hand sanitizer in their hands, I mean I ask them to use, they have a perception that I am a very clean person as well, and they always feel safe with me as well, you know?

Participant 17; aged 33 years; 2 years in the sex industry; brothel based

## Discussion

### Principal Findings

This mixed methods study identified risk factors for oropharyngeal gonorrhea among sex workers in Australia’s 2 most populous cities, and it provided some explanations for the factors and forces that underpin oral sex and mouthwash practices. Our findings show that performing condomless fellatio on clients is associated with oropharyngeal gonorrhea. Furthermore, sex workers frequently tongue kiss clients as previously reported [16,41], yet this practice was not a significant risk factor for oropharyngeal gonorrhea. From our interviews with sex workers in Melbourne, almost all reported kissing clients due to demand for “the girlfriend experience” and generally reported following the client’s lead with regard to kissing style and duration. However, they used condoms for fellatio, often citing that it is illegal not to in Victoria, and they considered condomless fellatio a risky practice for contracting STIs, unlike cunnilingus without a dental dam. Client saliva use as a lubricant was similarly often viewed as risky and “gross” even among those who engaged in cunnilingus with clients without a dental dam.

Our study showed high proportions of condom use among sex workers for fellatio with clients. Previous studies have reported that 79% [16] of female sex workers in Melbourne and 75% of female sex workers in Sydney used condoms for all fellatio activities with clients in an average working week [35] in the previous 3 months. It is possible that sex workers who are not attending sexual health clinics routinely may practice condomless fellatio more often with clients. One study from Sydney in 2017 examining advertisements for private sex work found that half of the female sex workers with web-based profiles were offering condomless fellatio (170/339, 50.2%) [42]; however, it is not clear how often these sex workers actually practice condomless fellatio in an average working week.

In our multivariable analysis, kissing was not a significant risk factor for oropharyngeal gonorrhea; however, it should be noted that the adjusted OR of having oropharyngeal gonorrhea was 1.5 (95% CI 0.7-3.5) among sex workers who tongue kissed male clients compared to those who did not tongue kiss male clients. The OR was >1 even though it was not statistically significant, suggesting that kissing could be a potential risk factor for oropharyngeal gonorrhea and may have some clinical and public health implications. The non–statistically significant result may be due to the limited sample size to have sufficient power to detect the difference as the study was ceased earlier due to the COVID-19 pandemic. Given the overlapping nature of sex practices, particularly kissing, this is a common issue when determining risk factors for oropharyngeal gonorrhea [9]. It is interesting to note that the one case who was interviewed for the qualitative study had only reported tongue kissing clients in the previous 2 weeks as a risk factor for oropharyngeal gonorrhea as she did not have condomless fellatio with clients or engage in other practices with clients that involved saliva and she had no noncommercial sexual partners. However, previous research has found kissing to be a risk factor for oropharyngeal gonorrhea among men who have sex with men, and performing fellatio was not [8]. This is likely due to differences in testing practices and risk perceptions among men who have sex with men compared to heterosexual men, who are more likely to be clients of the sex workers in our study.

There have been previous case reports and epidemiological studies suggesting that tongue kissing can transmit oropharyngeal gonorrhea [8,43,44]. It is also interesting that this participant was diligent about using and having her clients use a particular cocktail of mouthwash (Betadine and Cepacol) before each booking, and after the booking in her case, because she was afraid of catching a cold from a client and having to take time off work. While investigations are being made into the role of Listerine [22,23], no studies to our knowledge have investigated the role of antibacterial mouthwashes such as Betadine and Cepacol in the transmission of gonorrhea. It is possible that the mouthwash cocktail she was using with her clients altered her oral microbiome in an unfavorable way and increased her odds of contracting gonorrhea. A previous study examining the incidence of syphilis among 96 men who have sex with men in Indonesia (2019) found that using antibacterial mouthwash containing chlorhexidine increased the odds of syphilis acquisition, and it was similarly suggested that this could be due in part to changes in the oral microbiome [45]. An Australian clinical trial has shown that daily use of Listerine or Biotene for 12 weeks had no significant effect on the oral microbiome [46]; however, no study to our knowledge has examined the effect of Betadine and Cepacol used in combination on the oral microbiome. In any event, it is clear from our interviews that, for some sex workers, mouthwash use gives the feeling of protecting against bacteria and viruses and is thus used as a safety precaution (in addition to being widely used for hygiene purposes), therefore establishing that the role of mouthwash use with regard to gonorrhea prevention is important to inform best practice.

Our findings from the case-control study that frequent mouthwash use was not associated with oropharyngeal gonorrhea positivity are consistent with those of a previous study among men who have sex with men that found no association between using any mouthwash daily and oropharyngeal gonorrhea positivity [47]. The duration and method of using mouthwash (ie, rinsing or gargling) can vary between individuals, and it remains to be seen whether duration and method could influence the ability of mouthwash to reduce the amount of gonorrhea bacteria in the oropharynx. Among a study of at-risk populations for oropharyngeal gonorrhea, female sex workers used mouthwash for the shortest duration (median 14 seconds); however, this was not a significant difference [48]. Future recommendations with regard to mouthwash use in this population, should it be found to be beneficial for reducing gonorrhea transmission, should take into account duration and method of use.

Our results showed no significant difference between controls and cases in the number of clients on whom they performed fellatio. A previous case-control study among men who have sex with men in Melbourne (2018) found that the number of casual partners in the previous 3 months was an independent risk factor for oropharyngeal gonorrhea [9]. When limiting our analysis to only those sex workers who reported performing fellatio on clients (excluding those who did not perform this activity in an average working week) during an average working week, cases had fewer clients with whom they engaged in fellatio (median 5 for cases; median 7 for controls; *P*=.01; data not shown in the *Results* section). It is possible that female sex workers who perform condomless fellatio on clients see fewer clients in an average working week given that they may make more money per client offering this service compared to only offering fellatio with a condom as some participants in the qualitative interviews reported being offered more money by clients to perform “natural” or condomless fellatio (though none of our participants reported accepting these offers, in part due to concerns over STI transmission). It is also possible that having fewer clients is part of a risk reduction strategy among female sex workers who perform condomless fellatio. Further research is required to investigate why female sex workers who offer condomless fellatio might perform fellatio on fewer clients than those who do not.

There was a higher proportion of cases who reported tongue kissing clients in an average working week compared to controls (51/60, 85% of cases tongue kissed clients in an average working week compared to 348/494, 70.4% of controls). However, in contrast to performing fellatio on clients, among those who said yes to kissing clients, there were no differences in the number of clients that cases tongue kissed in an average working week compared to controls. In our study, there were 60.1% (399/664) of female sex workers who answered yes to kissing clients in an average working week, which is lower than what a cross-sectional survey of female sex workers from the MSHC in 2018 found (83.7%) [16]. This could be in part due to recruitment for this study occurring at 2 sites and including a higher proportion of non-Australian female sex workers as the 2018 study showed that Asian language–speaking female sex workers were significantly less likely than English-speaking female sex workers to tongue kiss clients [16]. Female sex workers recruited from the SSHC and those born in China, Thailand, or other countries were less likely than those recruited at the MSHC and those from Australia or New Zealand to report tongue kissing (data not shown).

For some participants in our qualitative interviews, kissing and cunnilingus were the only sexual practices that might be influenced by how much pleasure the sex worker was experiencing during the booking, though pleasure impacting these behaviors was generally reported as a rare occurrence. These participants described kissing for longer or deeper if they were enjoying the booking. However, most of the participants in our study described experiencing pleasure at work as an infrequent or nonexistent occurrence. There has been limited research into pleasure for sex workers during sex work. A previous qualitative study of 9 female sex workers in Victoria explored this concept and reported that, for some women, sexual pleasure was possible with a client only after developing intimacy through seeing them multiple times; however, this study was exploratory and specifically recruited women who had positive experiences of sex work [49]. Further research could clarify the extent to which sex workers experience sexual pleasure at work and whether this impacts sexual practice.

The main limitation to this study is that most of the cases were recruited from one clinic (SSHC) and epidemiology may vary due to environmental and spatial factors. There was also a noted delay in recruiting cases at MSHC compared to SSHC, which could indicate a higher rate of declining to participate. One reason for this may be the varying laws regarding sex work in the state of Victoria versus NSW, whereby performing condomless oral sex was illegal in Victoria but not in NSW. This may have been a deterrent for taking part in this study as participants may not have felt comfortable reporting any sex practices that were illegal. Previous research has shown that regulated and criminalized sex work often discourages female sex workers to seek health care [50], so it could be that they are less likely to disclose their sex practices to clinicians, which would make it difficult to assess risk. Despite a slower recruitment of cases from the MSHC, among those sex workers who participated in the survey, participants from the MSHC were no more likely to decline to report sexual practices compared to those from the SSHC after adjusting for oropharyngeal gonorrhea diagnosis, age, and country of birth. Only being born in China or other countries aside from Thailand and New Zealand was associated with declining to report sex practices on the survey.

Another limitation of this study was that our convenience sampling may have created a bias toward those who attended sexual health clinics for HIV and STI screening (or presented with symptoms), and this may not be generalizable to the entire sex worker population, including those who did not attend a sexual health clinic. Most of our participants worked in brothels and massage parlors, and only 0.2% (1/664) participated in street-based sex work; thus, the findings may not be generalizable to those engaging in street-based sex work.

A final limitation of this study is that interviews for the qualitative component were only offered in Victoria due to the financial and logistic difficulties of the interviews being conducted only in English and by researchers based only at the MSHC. However, similar to all qualitative data, the qualitative component was not meant to be generalizable to the wider population of sex workers. Rather, this component of our study provided additional depth and understanding to the data collected in the quantitative component among Melbourne-based female sex workers. These interviews were also cut short due to the COVID-19 pandemic; however, sufficient meaning was generated from the data to answer the questions of clinical interest.

The availability of phone interviews in combination with face-to-face interviews can be considered a strength of our study as it allowed participants to freely disclose personal and sensitive information. We found that our interviews over the phone provided rich data that were comparable to, and in some cases deeper, than those from our face-to-face interviews. Allowing the option of phone interviews, particularly asking the participants to select a phone or face-to-face interview upon recruitment, likely encouraged a wider array of participants as it is possible that some participants who would have been uncomfortable with a face-to-face interview opted to share their experiences over the phone rather than declining to participate. Our findings in this case reflect those of other studies that have shown no difference in data quality between phone interviews and face-to-face interviews [51,52].

### Conclusions

Our study shows that condomless fellatio is a risk factor for oropharyngeal gonorrhea among sex workers despite most sex workers using condoms with their clients for fellatio. Novel interventions, particularly targeting the oropharynx, will be required for oropharyngeal gonorrhea prevention.

## Appendix

Multimedia Appendix 1 A map of the data overlaid with variables examined.

Multimedia Appendix 2 Recruitment flowchart.

Multimedia Appendix 3 Associations with declining to answer sexual practice questions in survey among 664 female sex workers.

## Abbreviations

AMR: antimicrobial resistance
HERS: Health Research in Sex Workers
MSHC: Melbourne Sexual Health Centre
NSW: New South Wales
OR: odds ratio
RhED: Resourcing Health & Education
SSHC: Sydney Sexual Health Centre
STI: sexually transmitted infection
SWOP: Sex Workers Outreach Project

## Data Availability

The data sets generated during and analyzed during this study are not publicly available due to ethics requirements considering the confidential nature of patient data. Further information can be obtained by contacting the Alfred Hospital Ethics Committee, Melbourne, Victoria, Australia (project 596/17).
