# Supplementary figures and images for: Understanding Risk Factors for Oropharyngeal Gonorrhea Among Sex Workers Attending Sexual Health Clinics in 2 Australian Cities: Mixed Methods Study
Source: JMIR Public Health Surveill. 2024 May 20;10:e46845. doi: 10.2196/46845 (PMC11148521; doi:10.2196/46845)

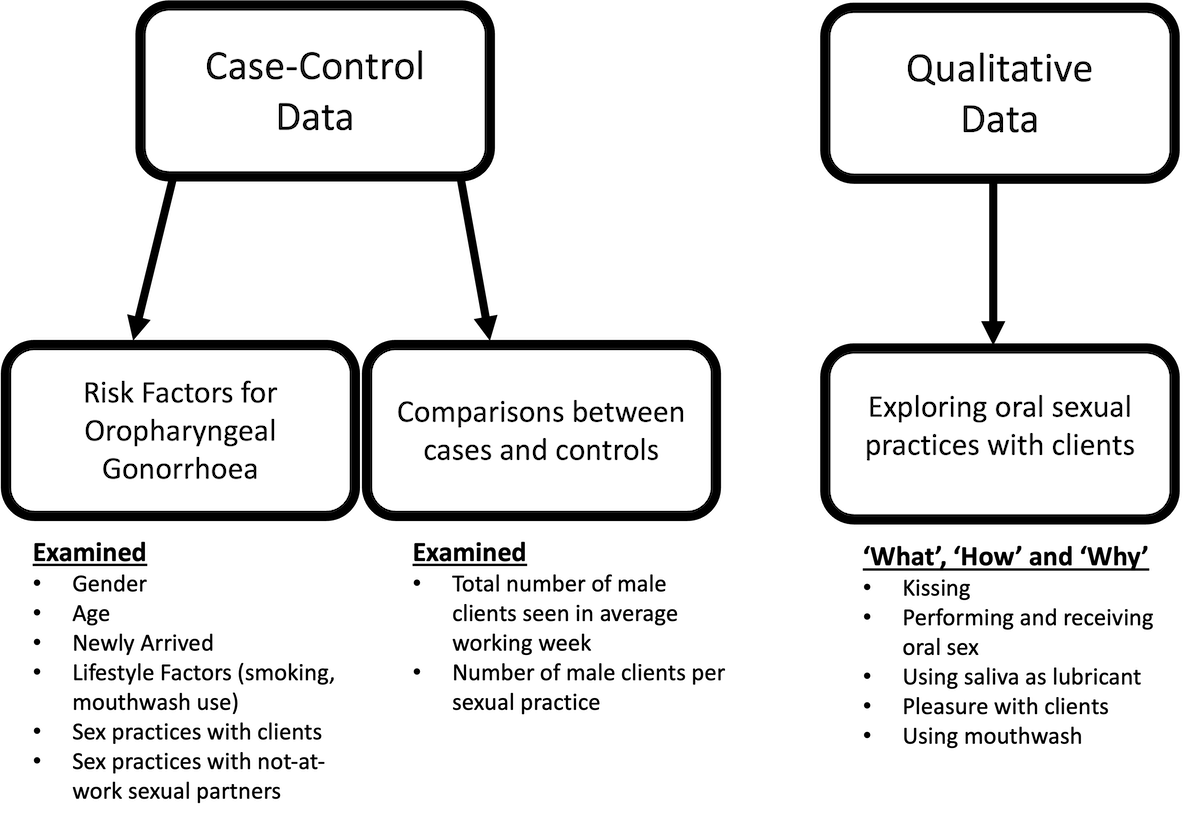

Supplement: Multimedia Appendix 1 [file publichealth_v10i1e46845_app1.png]

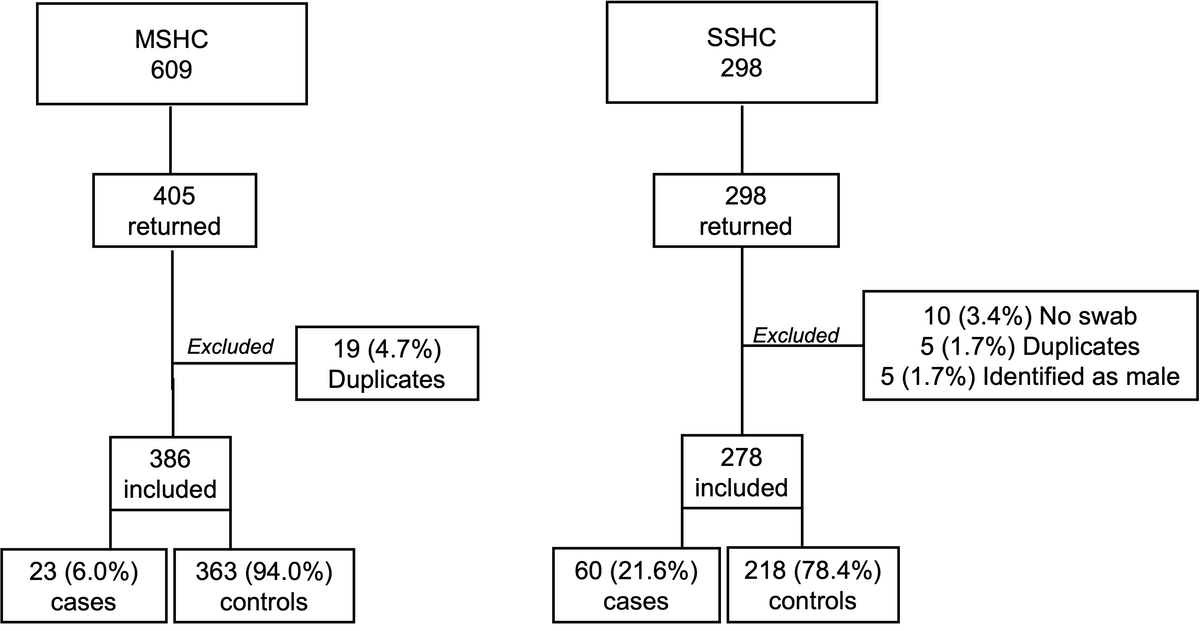

Supplement: Multimedia Appendix 2 [file publichealth_v10i1e46845_app2.png]
