# Supplementary material for: Understanding Risk Factors for Oropharyngeal Gonorrhea Among Sex Workers Attending Sexual Health Clinics in 2 Australian Cities: Mixed Methods Study
Source: JMIR Public Health Surveill. 2024 May 20;10:e46845. doi: 10.2196/46845 (PMC11148521; doi:10.2196/46845)
Supplement: Multimedia Appendix 3 [file publichealth_v10i1e46845_app3.docx]

Supplemental Table 1: Associations with declining to answer sexual practice questions in survey among 664 female sex workers

|  | Total number participating FSW | Incomplete section on sex practices ^A^(%) | OR (95% CI) | aOR (95% CI) |
| --- | --- | --- | --- | --- |
| Oropharyngeal gonorrhoea |  |  |  |  |
| Negative (control) | 581 | 90 (15.5) | ref | ref |
| Positive (case) | 83 | 20 (24.1) | 1.7 (1.0-3.0)* | 0.9 (0.5-1.6) |
|  |  |  |  |  |
| Site |  |  |  |  |
| MSHC | 386 | 37 (9.6) | ref | ref |
| SSHC | 278 | 73 (26.3) | 3.4 (2.2-5.2)* | 1.6 (0.8-3.2) |
|  |  |  |  |  |
| Age (years) |  |  |  |  |
| <=24 | 129 | 9 (7.0) | ref | ref |
| 25-34 | 332 | 46 (13.9) | 2.1 (1.0-4.5)* | 1.1 (0.5-2.5) |
| >=35 | 203 | 55 (27.1) | 5.0 (2.4-10.4)* | 1.9 (0.8-4.2) |
|  |  |  |  |  |
| Newly arrived to Australia (within 3 years) |  |  |  |  |
| No | 404 | 47 (11.6) | ref | ref |
| Yes | 260 | 63 (24.2) | 2.4(1.6-3.7)* | 0.8 (0.5-1.4) |
|  |  |  |  |  |
| Country of birth |  |  |  |  |
| Australia/New Zealand | 230 | 9 (3.9) | ref | ref |
| China | 156 | 57 (36.5) | 14.1(6.7-29.7)* | 9.3 (3.4-25.1)** |
| Thailand | 166 | 27 (16.3) | 4.8 (2.2-10.4)* | 2.7 (1.0-7.5) |
| Other Overseas | 112 | 17 (15.2) | 4.4 (1.9-10.2)* | 4.1 (1.7-9.7)** |

^A^Incomplete sections for sex practice with clients or not-at-work sexual partners.
